# Cholangiographic Features in Opium-Addicted Patients at a Tertiary Hospital in Iran

**DOI:** 10.1155/2012/510536

**Published:** 2012-10-15

**Authors:** Amir Houshang Mohammad Alizadeh, Esmaeal Shams Afzali, Azar Sanati, Anahita Shahnazi, Dariush Mirsattari, Mohammad Reza Zali

**Affiliations:** Gastroenterology and Liver Diseases Research Center, Shahid Beheshti University of Medical Sciences, Tehran, Iran

## Abstract

*Background/Aims.* Destructive and metabolic changes in hepatobiliary system have been demonstrated following opium use; however, cholangiographic features of bile ducts in opium-addicted patients with sphincter of Oddi dysfunction are not clearly determined. We described these differences and assessed the effects of opium use on postendoscopic retrograde cholangiopancreatography complications. *Methodology*. One hundred and nineteen patients with the diagnosis of sphinctre of Oddi dysfunction according to the Geenen-Hogan classification were studied. Eight patients were confirmed opium-addicted and others were nonaddicted. Change of serum amylase concentrations after endoscopic retrograde cholangiopancreatography and clinical diagnosis of addicted and non-addicted patients were compared. *Results*. Serum concentrations of liver aminotransferases and alkaline phosphatase were similar between the two groups. Serum concentration of amylase before endoscopic retrograde cholangiopancreatography was similar between them, whereas concentration of this enzyme was higher in nonaddicted ones after endoscopic retrograde cholangiopancreatography. Regarding pathologic changes in papilla, opium addiction group in comparison with control group statistically showed more tumoral features (25.0% versus 5.4%) and ulcerated changes (12.5% versus 0.0%). *Conclusions*. Opium use can increase probability of papilla ulcerative and tumoral changes in patients with sphinctre of Oddi dysfunction. Postendoscopic retrograde cholangiopancreatography serum amylase level may be reduced following opium addiction.

## 1. Introduction

Opium addiction with its harmful health, social, and security consequences remains one of the greatest threats to the well-being of humanity. It has been estimated that 13.5 million individuals suffered from opium addiction throughout the world and is increasingly prevalent among younger people in developing countries. In Iran, the prevalence of opium addiction has grown up by threefolds for the past twenty years, and it is presumed to be 2–2.8% [1]. The adverse consequences of opium use were clearly described as physical and psychological disability [2] and also as metabolic damage including aggravating effects on atherosclerosis formation related with hypercholesterolemia [3], potentially interfering with water and iron metabolism [4], and decreasing blood glucose [5]. Destructive and metabolic changes in hepatobiliary system have been also demonstrated in different stages of opium narcomania as considerable increase of liver enzymes activities [6] as well as dilated biliary ducts especially without any biliary symptoms [7]. Additionally, administration of opioid drugs could be resulted in bile duct obstruction and sphincter spasm during hepatobiliary scans and therefore, using these drugs before a diagnostic hepatobiliary procedures was not recommended [8–12]. However, a few studies are available in cholangiographic features of bile ducts in opium addicted and non-addicted patients with sphincter of Oddi dysfunction (SOD). This study described these differences and assessed the effects of opium use on post-ERCP complications.

## 2. Methodology

Between 2008 and 2011, 780 patients with calculus disease of the biliary tract and candidates for endoscopic retrograde cholangiopancreatography (ERCP) were evaluated by the liver services of Taleghani Hospital in Tehran. Included in the analysis were 8 opium addicted patients as the case and 111 non-addicted patients as the control groups with the diagnosis of SOD according to the Geenen-Hogan classification [13]. Opium addiction was defined according to the DSM-IV for substance dependence as daily regular use of opium [14]. All patients signed research study informed consent documents, and the study was approved by the ethics committee of the internal review board of Shahid Beheshti University of Medical Sciences.

Demographic characteristics, medical history, and mode of presentation were recorded by interviewing in the day of admission. Patients with a history of biliary sphincterotomy or precut sphincterotomy, preprocedure active pancreatitis, pregnancy, mental disability, and refusal to participate were excluded. The first liver function tests results during the acute admission used as the screening tests for ERCP. Serum amylase was also measured 30 minutes before and 3 hours after ERCP. All patients underwent ERCP for suspected and diagnosed pancreatobiliary disease and on the basis of generally accepted diagnostic indications for ERCP [15]. Procedure was performed under conscious sedation with midazolam and meperidine and by a staff gastroenterologist or by a fellow under direct supervision [15]. To determine the effect of opium addiction on characteristics of pancreatobiliary disorders and change of serum amylase concentrations after ERCP, ERCP reports and clinical diagnosis of addicted and non-addicted patients were compared.

Results were expressed as the mean ± standard deviation (SD) for quantitative variables and percentages for categorical variables. Categorical variables between the variables were compared using *χ* ^2^-square test or Fisher's exact test if required; continuous variables were compared by independent samples *t* test for variables with normal distributions and Mann-Whitney test for variables with nonnormal distributions. *P* values of 0.05 or less were considered statistically significant. All the statistical analyses were performed using SPSS version 16.0 for windows (SPSS Inc., Chicago, IL, USA).

## 3. Results

Male to female ratio was higher in opium-addicted than non-addicted patients. Regarding medical history (Table 1), cigarette smoking was more prevalent in addicted patients, and other risk factors such as hypertension, diabetes mellitus, and coronary artery disease were comparable in opium addicted and non-addicted patients. History of cirrhosis, bile stone, and pancreatitis were similarly found in the two groups. With regard to clinical manifestations, icterus was more observed in non-addicted than addicted patients. However, presence of other manifestations was similar between the two groups (Table 2). Among liver function parameters (Table 3), serum concentrations of AST and ALT and ALP were similar between them. Although serum concentration of amylase before ERCP procedure was similar between the two groups, concentration of this diagnostic enzyme was higher in non-addicted ones after ERCP.

Successful biliary cannulation was achieved in 100% of the patients in opium addiction group and in 87.1% of patients in another group (*P* = 0.279); however, cannulation was slightly more difficult in addiction group than other patients (33.3% versus 11.3%, *P* = 0.071). Regarding pathologic changes in papilla, opium addiction group in comparison with control group showed more tumoral features (25.0% versus 5.4%, *P* = 0.032) and ulcerated changes (12.5% versus 0.0%, *P* < 0.001). No significant differences were found in dilatation of hepatobiliary ducts between addicted and non-addicted groups. ERCP complications as post-ERCP pancreatitis, local bleeding, cholangitis, and GI perforation were not reported in opium addicted patients, but these events occurred in 4.5%, 0.9%, 2.7%, and 0.9% of non-addicted patients, respectively.

## 4. Discussion

The present study had some important findings. First of all, it was found that the rates of ulcerative and tumoral changes in papilla were notably higher in opium addicted patients than non-addicted ones. There is not a similar finding of related research in the existing medical literature, but some researchers have studied and confirmed the effects of opium use on other organs carcinoma. For instance, the relationships between opium addiction and bladder adenocarcinoma [16] and also gastrointestinal malignancies [17] were previously described. It can be hypnotized that the mechanism of opium addiction effects on malignant and ulcerative changes of hepatobiliary system is similar to other malignant-induced processes; Malaveille et al. studied the carcinogenic products in the opium pyrolysates and found that pyrolysates of opium, and particularly of morphine, a major opium alkaloid, were highly mutagenic substances [18]. Experimental studies have also shown that N-nitrousamine, which is a known carcinogen in the opium, alkylates the DNA of lung cells in mice and causes lung cancer [19]. In another study, abuse of opium dross (pyrolysis) has been shown to cause production of promutative compounds, which can cause cancer in addicted people who take opium dross orally [20]. However, more studies are needed to define precisely the patterns of hepatobiliary cancers incidence and their risk factors following opium consumption.

Secondly, we found that except for papilla pathological changes that were different in opium addicted and non-addicted patients, other ERCP features as ductal dilatation were similar between the two groups. Chuah et al. showed that opium addicts did get CBD pathology like non-addicts, and if indicated there should be no qualms about performing ERCP in them. However, in their study, the mean CBD diameter of addicted patients was significantly higher than the control group [7]. Besides, some other studies could show that chronic use of opium increased the sphincter Oddi tonicity [9, 12, 21, 22]. It seems that the effects of opiates on tonicity of hepatobiliary ducts are dependent on several factors such as duration of addiction [21], form of opium consumption as oral or inhaled, and concomitant consumption of other addicting substances.

Additionally and for the first time, we could show that opium addiction had reductive effect on serum amylase after ERCP. There are a few studies about the impact of opium on production and secretion of pancreatic amylase. In a study by Roberts-Thomson et al., patients given morphine as an opium alkaloid showed slightly increase in amylase plasma level [23]. In another study, it was shown that morphine reduced secretion of amylase by stimulating GTPase activity [24]. We also suggest that the effects of opium consumption can potentially influence patient's appetite and therefore, may affect the physiological processes of amylase secretion. However, further studies for highlighting this probable effect can be recommendable.

In conclusion, the present study had these major findings: (1) opium use can effectively increase probability of papilla ulcerative and tumoral changes in patients with SOD; (2) severity of hepatobiliary ductal dilatation is similar in opium-addicted and non-addicted patients; (3) post-ERCP serum amylase level can be reduced following opium addiction.

## Figures and Tables

**Table 1:** Baseline characteristics and medical history in opium-addicted and non-addicted patients with SOD and undergoing ERCP.

Characteristics	Opium addicted (*n* = 8)	Non-addicted (*n* = 111)	*P* value
Male gender	8 (100)	48 (43.6)	0.002
Age (years)	60.1 ± 16.9	57.8 ± 15.7	0.735
Diabetes mellitus	0 (0.0)	15 (13.5)	0.594
Hypertension	1 (12.5)	28 (25.2)	0.678
Coronary artery disease	1 (12.5)	8 (7.2)	0.478
Cigarette smoking	4 (50.0)	11 (9.9)	0.009
Alcohol use	0 (0.0)	5 (4.5)	0.999
Pre-ERCP	2 (25.0)	13 (11.7)	0.265
Biliary stones	1 (12.5)	8 (7.2)	0.478
Cirrhosis	0 (0.0)	1 (0.9)	0.999
Pancreatitis	0 (0.0)	4 (3.6)	0.999
Cancer of pancreas	1 (12.5)	1 (0.9)	0.130

**Table 2:** Clinical manifestations in opium-addicted and non-addicted patients with SOD and undergoing ERCP.

Manifestations	Opium addicted (*n* = 8)	Non-addicted (*n* = 111)	*P* value
Fever	0 (0.0)	6 (5.4)	0.999
Weight loss	2 (25.0)	39 (35.1)	0.713
Abdominal tenderness	4 (50.0)	31 (27.9)	0.232
Anorexia	2 (25.0)	31 (27.9)	0.999
Fatigue	0 (0.0)	6 (5.4)	0.999
Pruritus	0 (0.0)	37 (33.3)	0.056
Icterus	0 (0.0)	58 (52.3)	0.006
Dark urine	1 (12.5)	27 (24.3)	0.679
Diarrhea	0 (0.0)	3 (2.7)	0.999
Constipation	3 (37.5)	11 (9.9)	0.051

**Table 3:** Liver function parameters in opium-addicted and non-addicted patients with SOD and undergoing ERCP.

Laboratory parameters	Opium addicted (*n* = 8)	Non-addicted (*n* = 111)	*P* value
AST	31.3 ± 25.4	86.7 ± 73.6	0.051
ALT	53.7 ± 74.8	96.2 ± 94.2	0.194
ALP	637.0 ± 875.5	756.5 ± 524.0	0.733
Lactate dehydrogenase	383.3 ± 168.6	483.2 ± 372.1	0.424
Total bilirubin	2.3 ± 4.1	8.8 ± 10.2	0.096
Direct bilirubin	0.7 ± 1.4	4.8 ± 5.7	0.064
Pre-ERCP amylase	102.8 ± 62.1	185.7 ± 286.4	0.065
Post-ERCP amylase	258.3 ± 70.4	727.2 ± 126.2	0.006

## Abbreviations

SOD: Sphincter of Oddi dysfunction
ERC: Endoscopic retrograde cholangiopancreatography.
